# Luminescence resonance energy transfer between genetically encoded donor and acceptor for protein-protein interaction studies in the molecular chaperone HSP70/HSP90 complexes

**DOI:** 10.1038/s41598-018-21210-6

**Published:** 2018-02-12

**Authors:** Kaushik Bhattacharya, Lilia Bernasconi, Didier Picard

**Affiliations:** Département de Biologie Cellulaire, Université de Genève, 30 Quai Ernest-Ansermet, Sciences III, 1211 Genève 4, Switzerland

## Abstract

Complex patterns of protein-protein interactions (PPInts) are involved in almost all cellular processes. This has stimulated the development of a wide range of methods to characterize PPInts in detail. Methods with fluorescence resonance energy transfer can be technically challenging and suffer from several limitations, which could be overcome by switching to luminescence resonance energy transfer (LRET) with lanthanide ions such as Tb^3+^. With LRET, energy transfer between PPInt partners works over a larger distance and with less topological constraints; moreover, the long-lived luminescence of lanthanides allows one to bypass the short-lived background fluorescence. We have developed a novel LRET method to investigate PPInts between partners expressed as fusion proteins with genetically encoded donor and acceptor moieties. Upon UV excitation of a tryptophan within a lanthanide binding peptide, the Tb^3+^ luminescence is harnessed to excite either a green or a red fluorescent protein. We demonstrate the usefulness of the LRET assay by applying it to analyze the interactions of the molecular chaperones HSP70 and HSP90 with their common co-chaperone HOP/Sti1. We recapitulate the previously described interaction specificities between the HSP70/HSP90 C-termini and tetratricopeptide repeat domains of HOP/Sti1 and demonstrate the impact of single point mutants on domain-domain interactions.

## Introduction

A detailed knowledge of protein complexes and protein-protein interactions (PPInts) is essential to understand cellular processes. Amongst the large panel of methods that have been developed to investigate PPInts both qualitatively and quantitatively with purified components or in living cells, some are based on Fluorescence Resonance Energy Transfer (FRET). Luminescence Resonance Energy Transfer (LRET) is a potential alternative to FRET where the donor molecule is a luminophore instead of a fluorophore, whereas the acceptor molecule is still a fluorophore. LRET^1–5^ with the luminescence of lanthanide ions could overcome several shortcomings of standard FRET because (i) the Förster radius is larger, allowing energy transfer over a larger distance; (ii) the donor emission is not polarized as in FRET, which provides for a greater topological flexibility between donor and acceptor; (iii) the luminescence of lanthanides does not bleach and is long-lived (up to the millisecond range)^6^; thus, the short-lived (up to nanoseconds) background fluorescence, which is due to the direct fluorescence of the acceptor, a common issue with FRET, can therefore be circumvented with a time gate of 10–100 µsec.

The difficulty with lanthanide ions is that they must somehow be chelated for excitation and for “attachment” to one of the interaction partners. Imperiali and colleagues first demonstrated that the luminescence of the lanthanide ion Tb^3+^ can be used as a donor signal when it is complexed by a short lanthanide binding polypeptide tag (LBT)^1,3,7^. LBTs are short and genetically encodable polypeptide sequences derived from Ca^2+^ binding loops of various proteins such as troponin C. Since lanthanide ions have similar ionic and structural characteristics as Ca^2+^ ions, they have a significant binding affinity for Ca^2+^ binding loops^8^. The affinity of lanthanide ions to LBTs can be in the low nM range. LBTs have been used with the lanthanides Tb^3+^ and Eu^3+^ for various biochemical and structural studies including LRET, Nuclear Magnetic Resonance, X-ray crystallography^1,9,10^. For LRET assays, the lanthanide luminescence can be induced with UV at 280 nm because of a properly positioned tryptophan residue in the LBT.

While LRET with LBT-lanthanide complexes can easily be set up for experiments in cell-free systems, applying it to intracellular PPInts in mammalian cells is more challenging because of the excitation wavelength (280 nm) and cellular Tb^3+^ uptake. Indeed, Tb^3+^ may not passively cross mammalian cell membranes and apparently cannot use a specific ion channel either. Therefore, this particular version of the technique with LBTs has so far only been applied to mammalian cells to investigate interactions outside of the cells, notably the structural rearrangements of a Na^+^/K^+^ ATPase pump and of a large-conductance Ca^2+^-and voltage-activated K^+^ channel with fluorescently labeled ouabain and iberiotoxin as acceptors, respectively^11,12^. Similar experiments have been done in bacterial cells to study the interaction between membrane-bound oligosaccharyl transferase and its substrates^13^.

These proof of concept experiments had established LBTs as useful components of LRET assays, but they had not yet been combined with fluorescent proteins as acceptors to investigate PPInts of full-length proteins or individual domains. We decided to implement LRET for these types of studies with two major molecular chaperone machines and their common co-chaperone HOP as the model system. Molecular chaperones are essential both in normal and stressed conditions; they ensure protein folding, integrity and stability, and homeostasis^14–16^. The core components of two of the major molecular chaperone systems are the Heat Shock Protein 90 (HSP90) and Heat Shock Protein 70 (HSP70)^17–21^. HOP, also known as STIP1 in mammals, is a co-chaperone, which acts as an adaptor molecule binding HSP90 and HSP70 simultaneously, and thereby facilitating substrate transfer from HSP70 to HSP90 in the process of protein folding and maturation^22–25^.

We chose the binary interactions within the ternary complex HSP70-HOP-HSP90, which is conserved in eukaryotes, to develop our “fit for purpose” LRET assay. We encoded the LBT and fluorescent proteins genetically in the two partner proteins as donor and acceptor, respectively, and tested their interactions *in vitro*. These experiments demonstrated that this LRET assay can easily be applied to PPInts with recombinant proteins and that multiple emission peaks of the lanthanide ion can be exploited for “multicolor” LRET.

## Results and Discussion

### LRET between a peptide-bound lanthanide ion and fluorescent protein as a readout for protein-protein interactions

**Figure 1:**
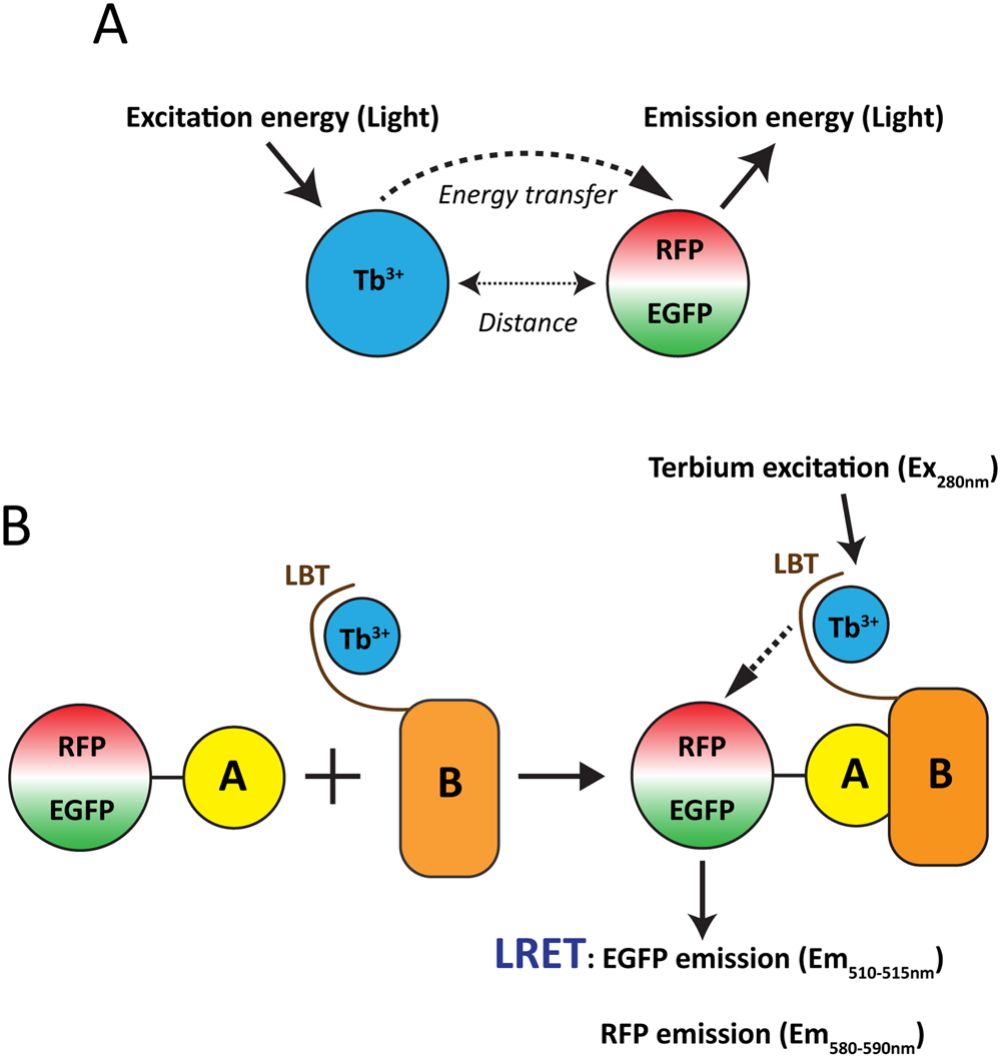
LRET schemes. (**A**) Schematic representation of the energy transfer process between donor (Tb^3+^) and acceptor (EGFP/RFP) molecules. (**B**) Experimental setup for the LRET PPInt scheme for interaction partner proteins A and B, one tagged with an LBT (donor molecule with the bound Tb^3+^) and another with EGFP or RFP (acceptor molecule). Relevant wavelengths for excitation (Ex) and emission (Em) are indicated.

**Figure 2:**
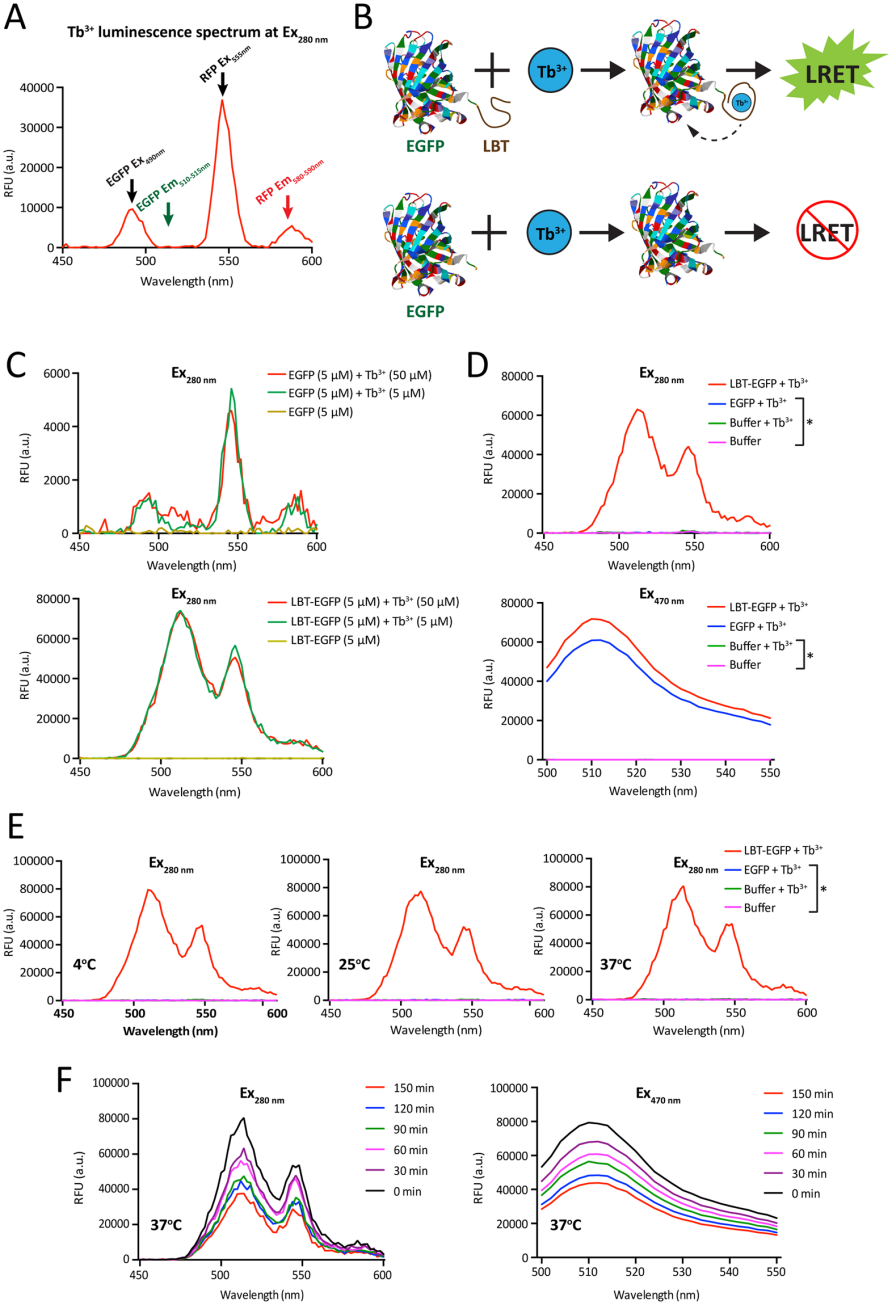
A proof of concept of intramolecular LRET. (**A**) Tb^3+^ luminescence spectrum upon excitation at 280 nm. The first Tb^3+^ emission peak at 490 nm corresponds to the EGFP excitation maximum and the second at 544 nm to the RFP excitation maximum. Their respective emission peaks (Em) are indicated with a green and red arrow. (**B**) Scheme of the intramolecular LRET assay with LBT-EGFP. (**C**,**D**) LRET-mediated EGFP fluorescence is dependent on LBT, Tb^3+^ (**C**) and EGFP (top graph in panel **D**). Excitation was at 280 nm with a 100 μs time delay. Intrinsic EGFP fluorescence measured without any time delay is independent of LBT and Tb^3+^ (bottom graph in panel **D**); the asterisk in panel **D** points out the lines that indistinguishably overlap at the level of the X-axis. (**E**) LRET assay at different temperatures; the concentrations were 5 µM; the asterisk points out the lines that indistinguishably overlap at the level of the X-axis. (**F**) LRET and intrinsic EGFP signals over time with equimolar reaction mixtures (5 μM each) of LBT-EGFP and Tb^3+^.

To establish a novel flexible method based on LRET to analyze PPInts *in vitro*, we set out to develop the appropriate toolbox. The specific luminescence emission spectrum of lanthanide ions excited by UV at 280 nm can be harvested to induce green and red fluorescent proteins such as EGFP and RFP, green and red fluorescent BODIPY dyes and others (Fig. 1A)^1,26^. To use LRET for PPInt analyses, the lanthanide ion must somehow be attached to one of the interaction partners and the quantum yield of the emission must be boosted. These requirements can both be met with a genetically encoded LBT; we selected the LBT with the sequence YIDTNNDGWYEGDELLA because of its relatively high affinity for Tb^3+^ of 57 nM^8,27^. As acceptor, we decided to encode EGFP or RFP as fusion proteins with the other interaction partner and to evaluate their association with the LBT fusion protein by monitoring LRET in the presence of Tb^3+^ with recombinant proteins *in vitro* (Fig. 1B). Upon excitation at 280 nm, the luminescence of Tb^3+^ is characterized by major peaks at 490 nm, 544 nm, and 590 nm. The Tb^3+^ emission peak at 490 nm overlaps with the excitation peak of EGFP and the one at 544 nm can excite the monomeric TagRFP^28^, a modified RFP (Fig. 2A).

### Intramolecular LRET assay

For our initial characterization of the LRET system, we generated a probe for intramolecular LRET. We produced recombinant EGFP with and without an N-terminal LBT (Fig. 2B). By titrating LBT-EGFP and EGFP with Tb^3+^, we found that there is no energy transmission with EGFP alone, since the spectrum is similar to the spectrum of Tb^3+^ alone (Fig. 2C). In contrast, UV stimulation of LBT-EGFP generates an emission peak characteristic of EGFP (510–515 nm) (Fig. 2C). We also noticed that the LRET signal of LBT-EGFP is saturated with equimolar quantities of Tb^3+^ (Fig. 2C). An excess of Tb^3+^ ions did not yield any additional stimulation (Fig. 2D), consistent with the previously reported 1:1 LBT-Tb^3+^ stoichiometry^1,29^. UV at 280 nm does not induce an appreciable EGFP emission, whereas this is readily observed for both recombinant proteins with an excitation at 470 nm, even without the time delay between excitation and recording of the emission (Fig. 2D). We checked the stability of the LRET signal in our experimental system at different temperatures (4 °C, 25 °C and 37 °C) and found that this range of temperatures does not affect LRET (Fig. 2E). At 37 °C, the LRET signal is at least as stable as the intrinsic EGFP fluorescence directly induced at 470 nm, both of which decrease over time (Fig. 2F). These preliminary experiments suggested that it might be possible to apply the same tools to analyze the interaction between two different proteins.

### Intermolecular LRET assay

**Figure 3:**
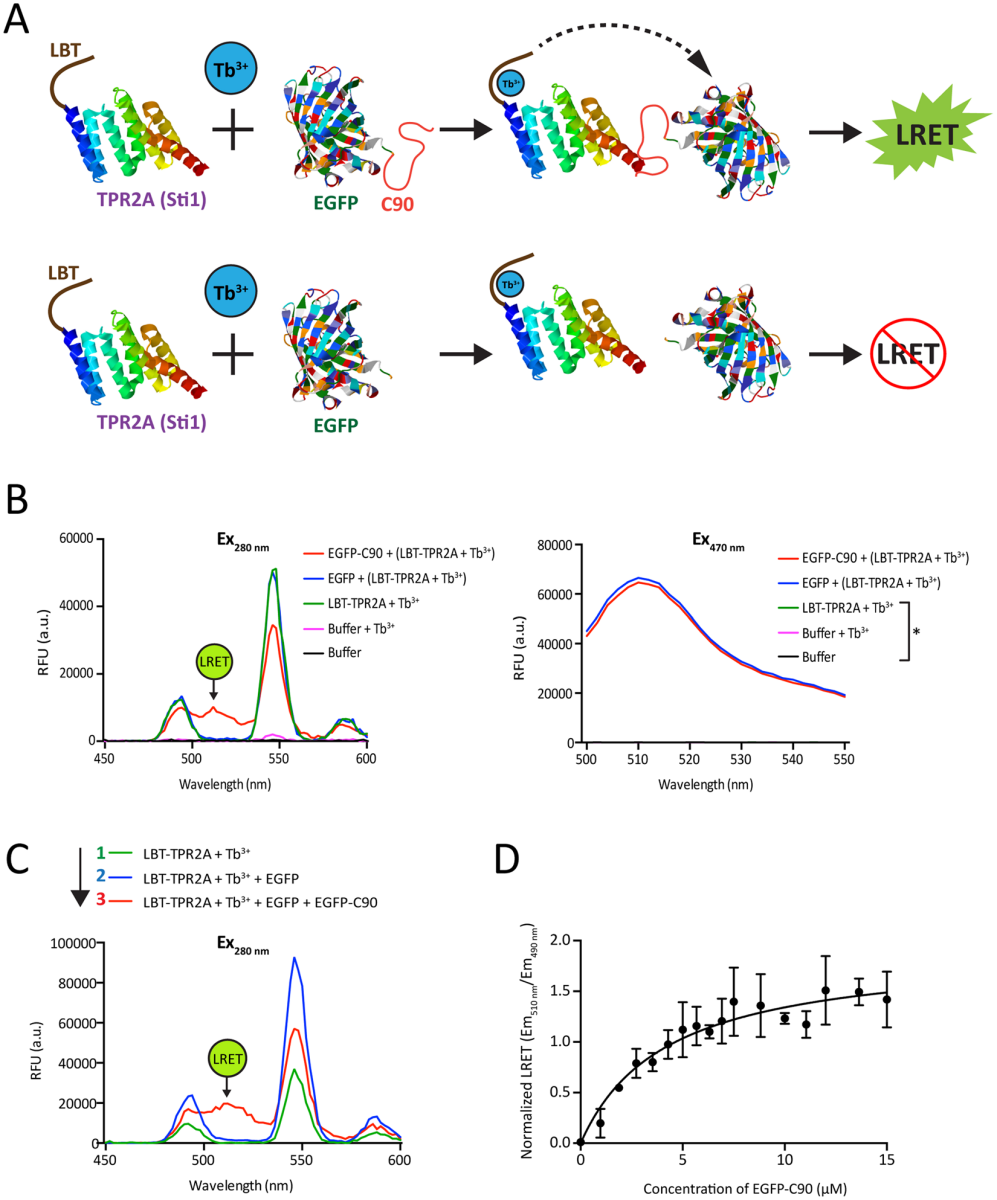
A proof of concept for the intermolecular LRET assay. **(A**) Scheme of the LRET assay with two interacting proteins. Here the interaction is between the very C-terminal MEEVD motif of HSP90 and the TPR2A domain of yeast Sti1. TPR2A was tagged with an N-terminal LBT and the C-terminal 90 amino acids of HSP90 (C90) were fused to EGFP. (**B**) LRET (left panel) and EGFP fluorescence (right panel) profiles of protein and buffer mixtures as indicated. Concentrations of proteins and Tb^3+^ were 5 µM. The position of the LRET signal is indicated and, on the right, an asterisk points out the lines that indistinguishably overlap at the level of the X-axis. (**C**) LRET profiles of a single-well experiment with the stepwise addition of EGFP and EGFP-C90 to LBT-TPR2A in the presence of Tb^3+^. The position of the LRET signal is indicated. (**D**) Titration experiment to determine the K_D_ of the interaction with LRET. TPR2A (20 μM) loaded with equimolar Tb^3+^ was titrated with increasing concentrations of EGFP-C90 (0–15 μM). The K_D_ was determined to be 4.2 ± 0.89 μM from the Scatchard plot from three independent experiments and presented as mean ± SEM.

As a model PPInt, we selected an evolutionarily conserved interaction in the HSP70-HSP90 molecular chaperone system between the extreme C-termini of HSP70 and HSP90, and the tetratricopeptide repeat (TPR) domains of yeast Sti1 or mammalian ortholog HOP^30^. Sti1 or HOP contain three different TPR domains: TPR1, TPR2A and TPR2B^22^. TPR2A is a high affinity HSP90-binding domain, whereas TPR1 and TPR2B bind the HSP70 C-terminus with moderate affinity^22,24^. For LRET, we generated two recombinant proteins: TPR2A from yeast Sti1 with an N-terminal LBT and the last 90 amino acids of human HSP90α fused to the C-terminus of EGFP (EGFP-C90), knowing that the C-terminal pentapeptide MEEVD of HSP90 should interact with TPR2A (Fig. 3A)^22^.

Upon combining EGFP-C90 and LBT-TPR2A in the presence of Tb^3+^, we could detect the specific LRET signal as an extra peak in the appropriate region of the EGFP emission spectrum (510–515 nm) (Fig. 3B). With EGFP alone or with LBT-TRP2A by itself, this peak was not observed, and the intrinsic fluorescence of EGFP and EGFP-C90 induced at 470 nm was comparable (Fig. 3B). To further confirm the interaction specificity and selectivity of this method, we did an order of addition experiment (Fig. 3C). Starting with LBT-TPR2A complexed with Tb^3+^, only Tb^3+^ luminescence could be seen. Upon addition of EGFP to the same mixture, the emission spectrum induced by 280 nm did not change. When EGFP-C90 was finally added to the same mixture, the appearance of an LRET signal supported our conclusion that it is indicative of a specific interaction of C90 with TPR2A (Fig. 3C).

To quantitate the interaction affinity, LRET was measured upon titrating LBT-TPR2A-Tb^3+^ with increasing concentrations of EGFP-C90. We determined the K_D_ to be 4.2±0.89 μM (Fig. 3D), which is consistent with a K_D_ of 5–6 μM reported in the literature and obtained with a range of other methods^22,31^. These results indicate that our LRET assay system can be applied for both qualitative and quantitative PPInt measurements.

### Diversification of intermolecular LRET by using alternative acceptor molecules

**Figure 4:**
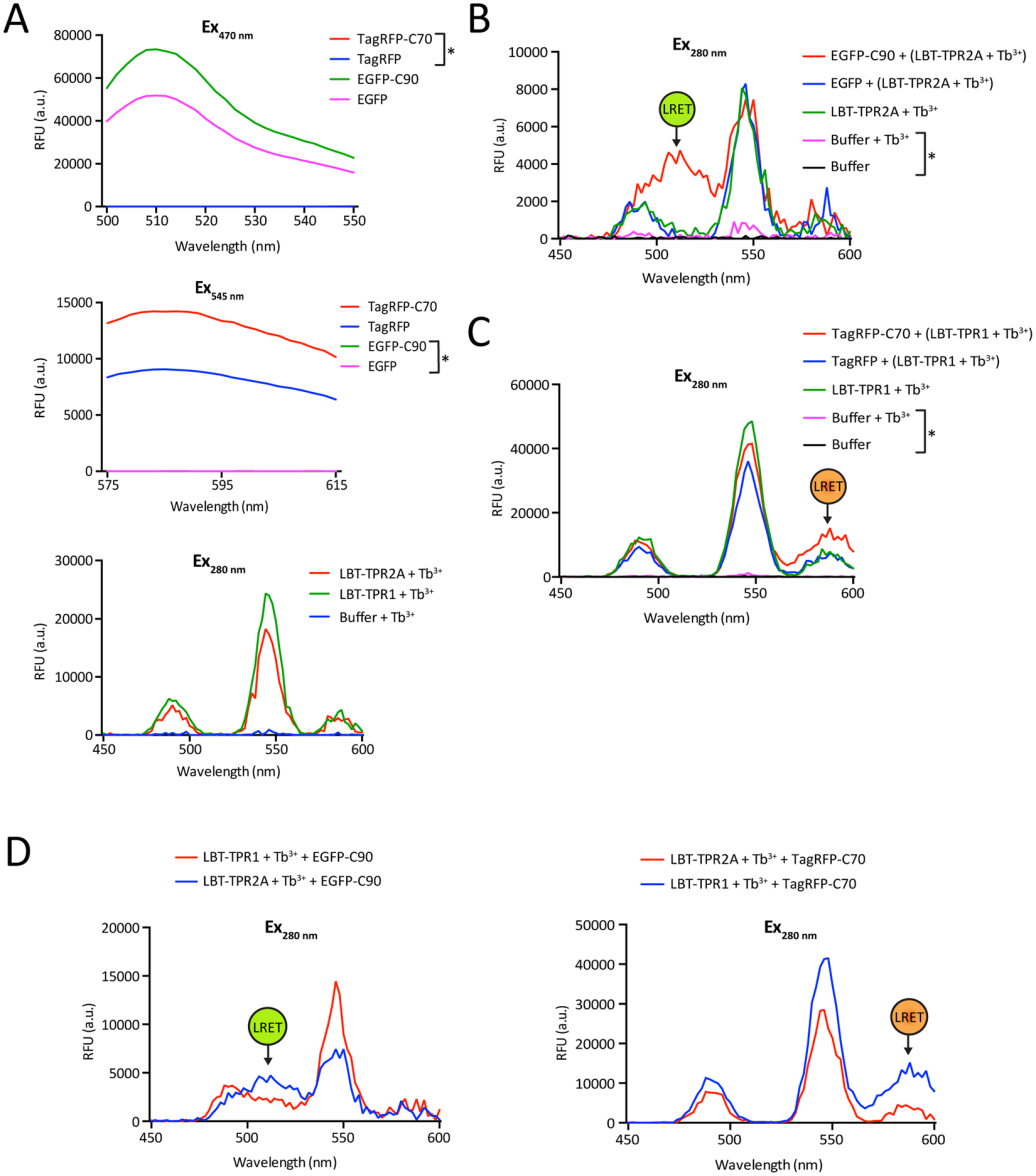
LRET assay with two different acceptors of the interaction specificity of individual TPR domains of human HOP. (**A**) Intrinsic fluorescence and luminescence as quality control of all the components used in the LRET assay. (**B**) LRET profile for the interaction between the C-terminal domain of HSP90 and the TPR2A domain of human HOP; proteins and Tb^3+^ were added at 5 μM. (**C**) LRET profile using the excitation of TagRFP as a readout for the interaction between the C-terminal domain of HSP70 and the TPR1 domain of human HOP; proteins were used at 25 µM. (**D**) LRET profiles confirm the specificity of the interactions of the individual TPR domains with either HSP90 or HSP70. Note that the data represented by the red line in panel C and the blue one in panel D are identical. In some panels, the position of the LRET signal is indicated and an asterisk points out the lines that indistinguishably overlap at the level of the X-axis.

To expand the range of our LRET method, we introduced an additional acceptor molecule along with EGFP. As mentioned earlier, the Tb^3+^ luminescence emission peak at 544 nm corresponds to the excitation peak of some red fluorescent proteins such as TagRFP. We decided to generate a TagRFP fusion protein with the extreme C-terminal domain of human HSP70 (C70), including the very C-terminal heptapeptide sequence PTIEEVD, which should allow it to interact with the TPR1 domain of human HOP^22^. Recombinant TPR1 protein, the interaction partner of C70 and also TPR2A, the interaction partner of C90, were generated with N-terminal LBTs. For quality control, the intrinsic fluorescence of EGFP and EGFP-C90 was checked by excitation at 470 nm, and that of TagRFP and TagRFP-C70 by excitation at 545 nm; the luminescence of the LBT-Tb^3+^ complex was checked with a time delay following excitation at 280 nm (Fig. 4A).

As before with the TPR2A domain of yeast Sti1, LRET could readily be monitored between LBT-TPR2A and EGFP-C90 (Fig. 4B). Similarly, the interaction of LBT-TPR1 with TagRFP-C70 could be monitored with the LRET signal in the region of the TagRFP emission maximum (580–590 nm) (Fig. 4C). This indicates that one can harness several peaks of the Tb^3+^ emission spectrum with an appropriate genetically encoded fluorescent protein as an acceptor molecule. With this PPInt determination system one could conceivably study interactions of three proteins at the same time. It should be noted, however, that while this may well work for qualitative assessments, the overlap of the TagRFP emission maximum (580–590 nm) and the Tb^3+^ luminescence peak (590 nm) may interfere with proper quantitation of that particular PPInt.

To check the specificity of our LRET assay further, we tested the cross-reactivity between the two TPR domains and the C-terminal domains of HSP90 and HSP70. LRET assays showed that TPR2A has a higher binding affinity for C90 and TPR1 for C70 at an equimolar ratio (Fig. 4D). Therefore, we can conclude that this LRET assay system is not only sensitive but that it also reports specifically and accurately on PPInts.

### LRET with HSP70 and HSP90 binding mutants of HOP

**Figure 5:**
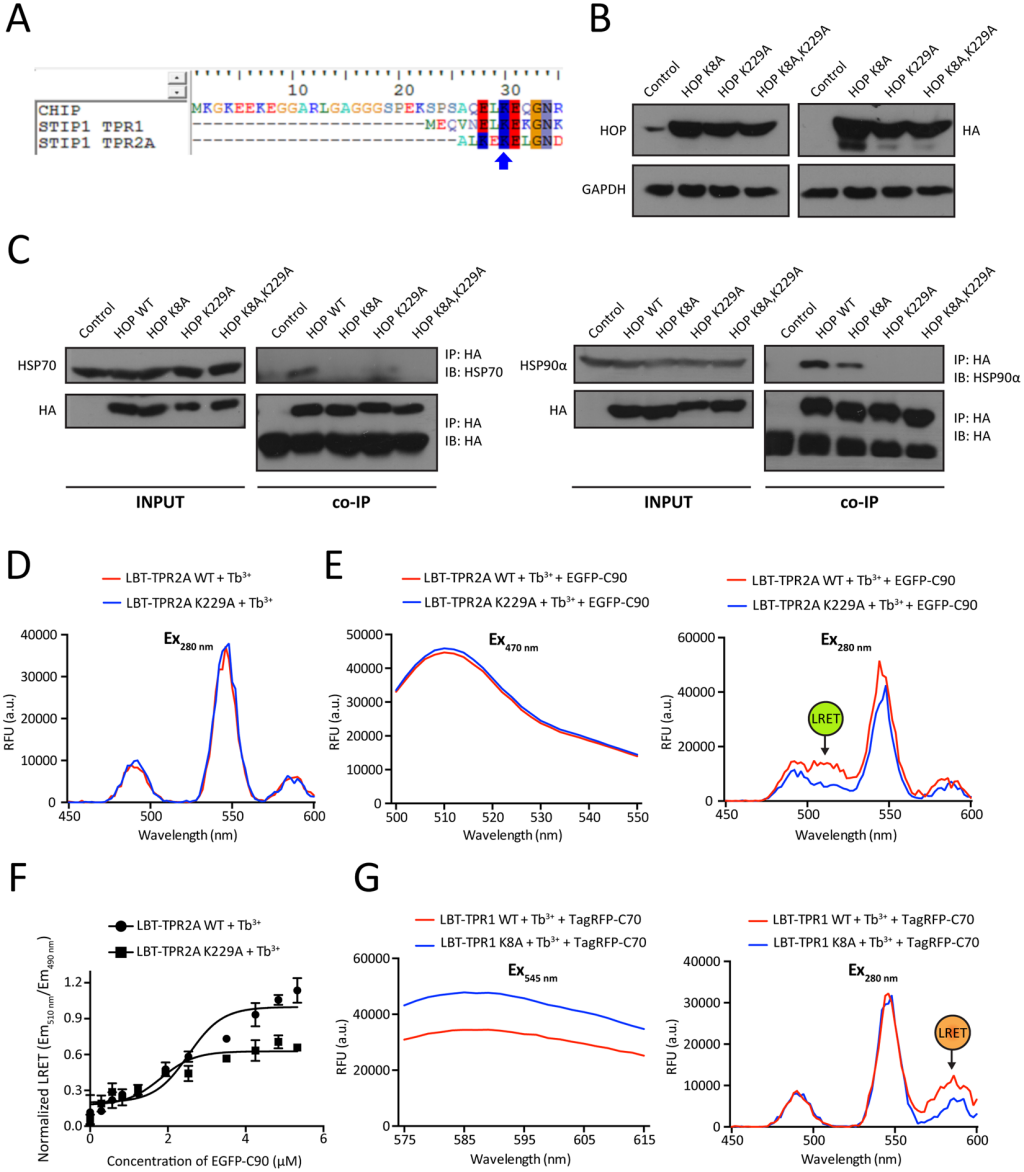
LRET assays of the interactions of TPR mutants of HOP with HSP70 and HSP90. (**A**) Sequence alignment of the relevant portions of the TPR of CHIP and of TPR1 and TPR2A of HOP; K30 of CHIP, which is known to be important for binding HSP70 and HSP90^32,33^, is highlighted with a blue arrow. (**B**) Immunoblot analysis of TPR point mutants; HA-tagged constructs were transiently expressed in HEK293T cells and revealed using both anti-HOP and anti-HA antibodies as indicated with GAPDH as loading control. (**C**) Co-immunoprecipitation experiments to check the association between HOP mutants and endogenous HSP70 and HSP90; IP, immunoprecipitation; co-IP, coimmunoprecipitation; IB, immunoblot with indicated antibody. The uncropped original images of the immunoblots shown in panels B and C are presented in Supplementary Fig. S2. (**D**) Luminescence patterns of Tb^3+^ bound wild-type (LBT-TPR2A WT) and point mutant (LBT-TPR2A K229A). (**E**) Intrinsic EGFP fluorescence and LRET profiles for wild-type (WT) and point mutant TPR2A. (**F**) LRET titration experiment comparing the binding of wild-type and mutant TPR2A to HSP90. TPR2A WT and K229A (10 μM) loaded with equimolar Tb^3+^ were titrated with increasing concentrations of EGFP-C90 (0–6 μM). The Scatchard plot of the normalized LRET from three independent experiments represents means ± SEM. (**G**) Intrinsic TagRFP fluorescence and LRET profiles for wild-type (WT) and point mutant TPR1. In some panels, the position of the LRET signal is indicated.

An LRET assay should be useful to investigate PPInt mutants. We therefore decided to generate TPR point mutants that would be defective for binding HSP70 and HSP90. To identify key residues, we aligned the sequences of the TPR domain of CHIP and TPR1 and TPR2A of mammalian HOP. Residue K30 of the TPR domain of CHIP has been shown to be the most important one for HSP70 and HSP90 binding^32,33^. As previously reported, we found a conserved lysine residue at this position in all three TPR domains^22^. Based on this, we could predict that K8 of TPR1 of HOP is important for HSP70 binding and K229 of TPR2A of HOP for HSP90 binding (Fig. 5A). This choice is supported by earlier results showing that the K8A and K229A mutants are unable to bind short C-terminal peptides of HSP70 and HSP90, respectively^34,35^, even though it must also be mentioned that the effect of K8A in mouse HOP has been reported not to affect the binding to HSP70 in NIH3T3 mouse fibroblasts despite its 98% sequence identity with human HOP^36^.

We generated the point mutants of these residues alone and in combination in the context of full-length human HOP. An immunoblot analysis of HEK293T cells transiently overexpressing the three mutants showed that they could be expressed at similar levels (Fig. 5B). To assess the association between wild-type and TPR mutants of HOP with HSP70/HSP90 *in vivo*, we used antibodies to the HA tag of HOP to determine the co-immunoprecipitation of HSP70 and HSP90α. The combined changes of K8A and K229A in HOP completely inhibit the binding of both HSP70 and HSP90α (Fig. 5C). The individual mutants K8A and K229A preferentially affect HSP70 and HSP90 binding, respectively. Interestingly, the individual mutants also reduce the binding of the correspondingly other molecular chaperone to the other TPR domain to some extent (Fig. 5C). We hypothesize that impairing HOP binding of HSP70 or HSP90 to its specific TPR domain prevents a hypothetical stretching or opening of HOP and as a result affects the interaction with the other molecular chaperone.

We then used the LRET assay to determine the impact of the mutants on binding the C-terminal domains of HSP70 and HSP90 *in vitro*. Tb^3+^ luminescence and intrinsic EGFP fluorescence were almost indistinguishable for wild-type and mutant TPR2A (Fig. 5D–G). In the LRET assay, the reduced binding of the TPR2A mutant K229A to EGFP-C90 could be observed both qualitatively (Fig. 5E) and quantitatively (Fig. 5F). Likewise, LRET with the TPR1 mutant K8A and TagRFP-C70 confirmed the reduced binding seen in the co-immunoprecipitation experiments (Fig. 5G). Hence, we conclude that our LRET assay faithfully monitors the impact of mutants on PPInts.

### Limitations of this LRET assay

In principle, LRET could be used in living cells, provided lanthanide-complexed proteins can be introduced into cells or generated in cells, and excited with a wavelength that is compatible with a living system. This had previously been accomplished by labelling a test protein with the lanthanide small-molecule chelator Lumi4, which allows the excitation of Tb^3+^ luminescence at 365 nm^5,26,37^. Encouraged by these results and our own success with *in vitro* experiments, we performed a series of pilot experiments towards porting the system to cells, even though we realized that an excitation at 280 nm would be difficult to implement with cells. We found that the Tb^3+^ luminescence itself and as a result LRET are strongly inhibited by several negatively charged and highly abundant intracellular molecules (ATP, PO_4_^3−^, and citrate) (see Supplementary Fig. S1A–C). This inhibition may be caused by the inherent insolubility of lanthanide phosphates and other lanthanide salts^38^. It is dose-dependent and ATP, for example, showed a strong inhibitory activity even at the lowest concentration tested (10 μM). This by itself would preclude *in vivo* experiments considering that the effective concentration of ATP in cells is in the mM range^39^. Additional abundant phosphate sources may include other nucleotides as well as free phosphate. Finally, we evaluated the inhibitory effects of crude cell lysates. Tb^3+^ luminescence is strongly inhibited by cell lysates even at very low concentrations (Supplementary Fig. S1D). These results point out that this particular version of the LRET assay is not appropriate for *in vivo* experiments. Because of these limitations, it also cannot be used for *in vitro* experiments that require these types of inhibitory compounds or chemicals.

### Conclusions

Our novel LRET assay for the analysis of PPInts is specific, sensitive, accurate, and quantitative. Provided the recombinant PPInt partners and a luminometer-fluorometer with time-gating are available, the assay is straightforward, cheap and fast. The effects of post-translational modifications (PTM), truncations, amino acid substitutions, and additional proteins on a particular PPInt can readily be assessed. For phosphorylation as a PTM, one could phosphorylate the protein before purification or mimic it with appropriate point mutants. Thus, this LRET assay does complement the existing toolbox for PPInt studies.

## Materials and Methods

### Materials and reagents

Anti-His (clone HIS-1) and anti-HA (HA.11) antibodies were obtained from Sigma. The antibodies to HSP90α (ADI-SPA-840, clone 9D2) and to HOP (ADI-SRA-1500, clone DS14F5) were obtained from Enzo Life Sciences, and to HSP70 (C92F3A-5) from StressMarq Biosciences. All the secondary HRP-coupled antibodies were purchased from Thermo Fisher Scientific.

### Plasmids

Plasmids pET/EGH and pET/LEGH allow bacterial expression of EGFP with a C-terminal His_6_-tag and LBT-EGFP with a C-terminal His_6_-tag, respectively. Coding sequences for full-length EGFP were also cloned into the XhoI site of the bacterial expression vector pET15b; this allowed the subsequent in-frame insertion of the coding sequences for the C-terminal 90 amino acids of human HSP90α (C90) into the BamHI site with an extra TAA termination codon for translation to terminate the protein sequence with MEEVD.

The coding sequences for TPR2A of yeast Sti1 (aa 252–379) were cloned along with sequences for the N-terminal LBT peptide (YIDTNNDGWYEGDELLA^8,27^) between NcoI and XhoI sites of vector pST96 (a gift from Caroline Gabus and Stéphane Thore, University of Geneva), a derivative of the pET family of vectors with coding sequences for a C-terminal His_9_-tag, engineering an EcoRI between the LBT and TPR2A sequences. To generate the master plasmid pST96/LBT-MCS with sequences for an N-terminal LBT followed by a polylinker, we cloned a polylinker between the EcoRI and XhoI sites of this plasmid pST96/LBT-TPR2A (Sti1), thereby eliminating the TPR2A coding sequences. We obtained the pcDNA3.1(+)-based expression plasmid for human HA-tagged HOP (STIP1) from Adrienne Edkins (Rhodes University, Grahamstown, South Africa). We performed site-directed mutagenesis to generate the human HOP mutants K8A (codon change: AAG > GCG), K229A (AAA > GCA), and their combination. Sequences for both wild-type and mutant TPR1 (aa 1–105) and TPR2A (aa 225–337) of human HOP were cloned into the XhoI site of plasmid pST96/LBT-MCS for bacterial expression of LBT and His_9_-tagged recombinant proteins.

TagRFP coding sequences were amplified from plasmid pTagRFP-C (Evrogen) as template and cloned into the BamHI site of plasmid pET45b; subsequently, the coding sequences for the 70 terminal amino acids (C70) of human HSP70 were cloned into the PstI site with an extra TAA termination codon to terminate the TagRFP-C70 protein sequence with PTIEEVD.

### Protein expression and purification

Protein expression was done with the *E. coli* Rosetta (DE3) pLysS strain. An overnight culture was diluted 75 to 100 times in fresh LB-medium and further cultured for 4 to 8 hrs, depending on the clone, until OD_600_ 0.5–0.7 with chloramphenicol and other antibiotics as appropriate. Cultures were cooled down and induced for protein expression by the addition of 0.5–1.0 mM IPTG at 25 °C–30 °C for 3–6 hrs, depending on the clone. Bacterial pellets from a large volume (1–2 liters) of induced culture were resuspended in 50 mM HEPES pH 7.0, 100 mM NaCl, 1× protease inhibitor cocktail (Thermo Fisher Scientific), and lysed with a French press. Cell debris and insoluble materials were discarded by centrifuging at 16,000×g for 20 minutes at 4 °C. Clarified bacterial cell lysates were applied to a Ni-NTA FPLC column or to a manually prepared Ni-NTA column. The unbound protein fraction was washed off with lysis buffer containing 20 mM imidazole, and elution of proteins specifically bound to the FPLC column was done with a 20 mM–500 mM imidazole gradient. Proteins were also eluted in bulk from Ni-NTA manual prepared columns with buffer containing 250 mM imidazole. Whenever possible, we dialyzed eluted proteins against 10 mM HEPES pH 7.2, 100 mM NaCl, 1 mM DTT and stored them in small aliquots with 10–20% glycerol at −80 °C. To avoid repeated freeze-thaw cycles, some samples were kept at 4 °C for as long as a few weeks.

### Tb^3+^ luminescence and LRET assays

TbCl_3_ was dissolved in 1 mM HCl at a final concentration of 50 mM as a stock solution. Working concentrations of the TbCl_3_ varied from 5 to 50 μM depending on the protein concentration used in the experiment. LRET assays were done with an equimolar mixture of two proteins with an excitation at a wavelength of 280 nm with a 100 μs time delay before recording the spectra. The Tb^3+^ luminescence and LRET experiments were done in 96-well black plates with a Synergy or Cytation 3 imaging reader (BioTek) with the following instrument settings: Fixed Excitation: 280/9.0 nm; Emission Start: 450/9.0 nm, Stop: 600 nm, Step: 2 nm; Optics: Top; Gain: 170–220; Time Resolved: Delay: 100 µsec, Collection Time: 300–1000 µsec; Read speed: Normal.

### K_D_ determination and LRET titration assays

To obtain K_D_ values, 20 μM of the LBT-TPR2A-Tb^3+^ complex was titrated with increasing concentrations of EGFP-C90. Normalized LRET was determined by calculating the ratio of the emission at 510 nm (Em_510 nm_) and the emission at 490 nm (Em_490 nm_) in arbitrary units (a.u.). In other experiments, the apparent binding difference between C90 and WT versus K229A of the TPR2A (STIP1) domain was determined by titrating the TPR2A-Tb^3+^ complex (10 μM) with increasing concentrations of EGFP-C90 and plotting the normalized LRET signal.

### Transfection of mammalian cells

HEK293T cells were transiently transfected with the polyethylenimine (PEI-MW-25000, Polysciences, Inc.) transfection reagent at a 1:4 DNA/PEI ratio.

### Protein extraction and co-immunoprecipitation

Control and transfected cells were harvested by scraping and washed with phosphate-buffered saline, centrifuged and resuspended in chilled lysis buffer (10 mM Tris-HCl pH 7.5, 50 mM NaCl, 1 mM EDTA, 1 mM DTT, 10% glycerol, 10 mM Na-molybdate and 1× protease inhibitor cocktail). Cells were lysed by sonicating for 30 seconds/cycle (total 30 cycles) at high power using a Bioruptor® sonicator (Diagenode). Cell debris were discarded by centrifugation and protein concentrations quantified by Bradford. For immunoprecipitation, 1 mg of total cell lysate was incubated with anti-HA antibodies overnight at 4 °C on a rotating wheel. Subsequently 25 µl of Dynabeads™-ProteinG (Thermo Fisher Scientific) were added and incubated for 3 hrs at 4 °C. Dynabeads were washed 6 times with chilled lysis buffer (with 0.1% Triton X-100) and collected with a magnetic stand. Beads were then boiled with a reducing sample buffer (containing 10 mM DTT) and retained with a magnetic stand to recover the protein containing eluate. Total cell extracts and eluates were used for SDS-PAGE and immunoblotting.

### Data availability

All data generated or analyzed during this study are included in this published article (and its Supplementary Information file).

## Electronic supplementary material

Supplementary information
