# Supplementary material for: Luminescence resonance energy transfer between genetically encoded donor and acceptor for protein-protein interaction studies in the molecular chaperone HSP70/HSP90 complexes
Source: Sci Rep. 2018 Feb 12;8:2801. doi: 10.1038/s41598-018-21210-6 (PMC5809404; doi:10.1038/s41598-018-21210-6)
Supplement: Supplementary file 1 — Supplementary information [file 41598_2018_21210_MOESM1_ESM.pdf]

Luminescence resonance energy transfer between genetically encoded donor and acceptor for protein-protein interaction studies in the molecular chaperone HSP70/HSP90 complexes

Kaushik Bhattacharya, Lilia Bernasconi, and Didier Picard

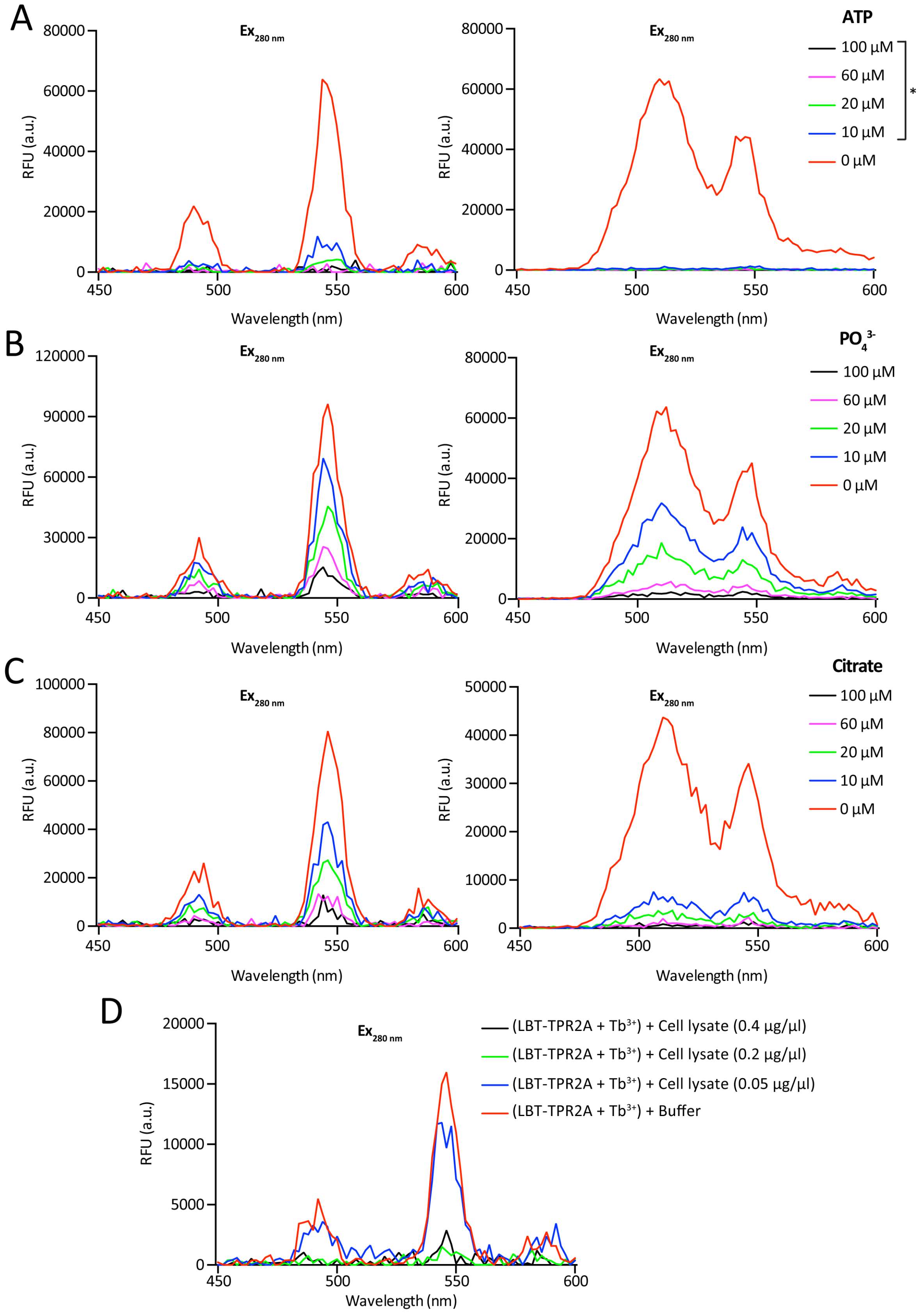

Figure S1. Limitations of the LRET assay. Tb^{3+} luminescence (with LBT-TPR2A) and intramolecular LRET (with LBT-EGFP) profiles of titration experiments with intracellularly abundant negatively charged molecules as indicated: (**A**) ATP, (**B**) PO_4^{3-}, and (**C**) citrate. (**D**) Tb^{3+} luminescence (with LBT-TPR2A) in the presence of increasing concentrations of mammalian cell lysates. In panel **A**, an asterisk points out the lines that indistinguishably overlap at the level of the X-axis.

HOP, HA and GAPDH in Fig. 5B

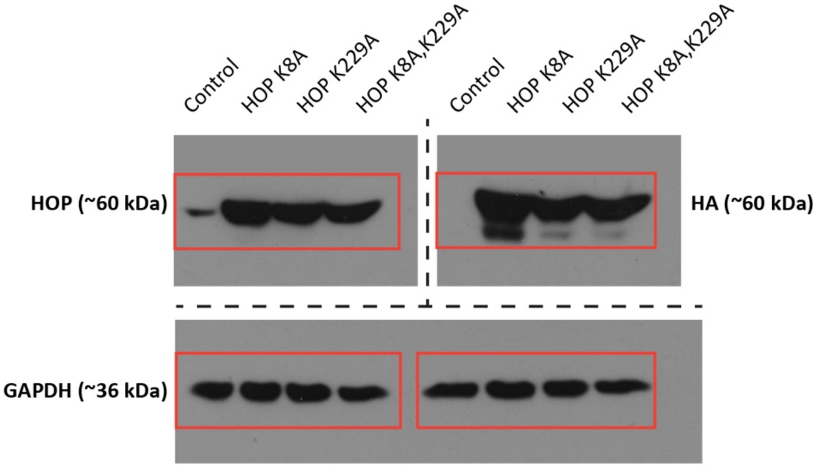

HA, HSP70 and HSP90α in Fig. 5C

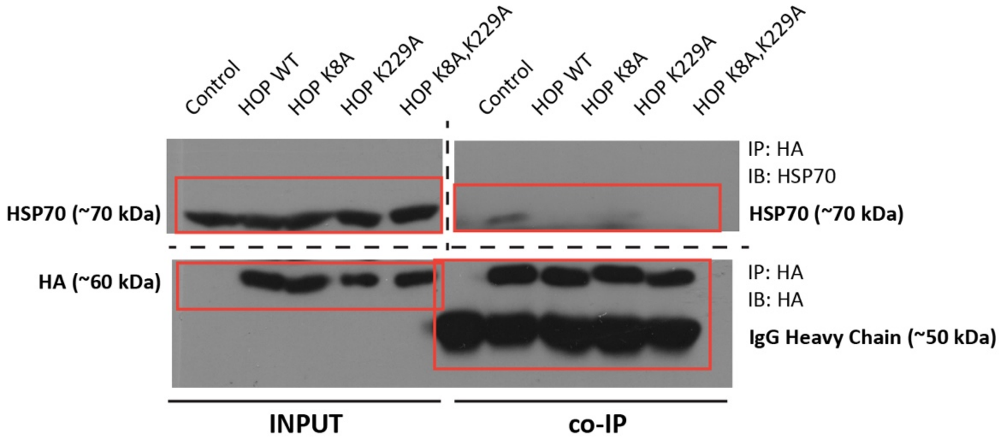

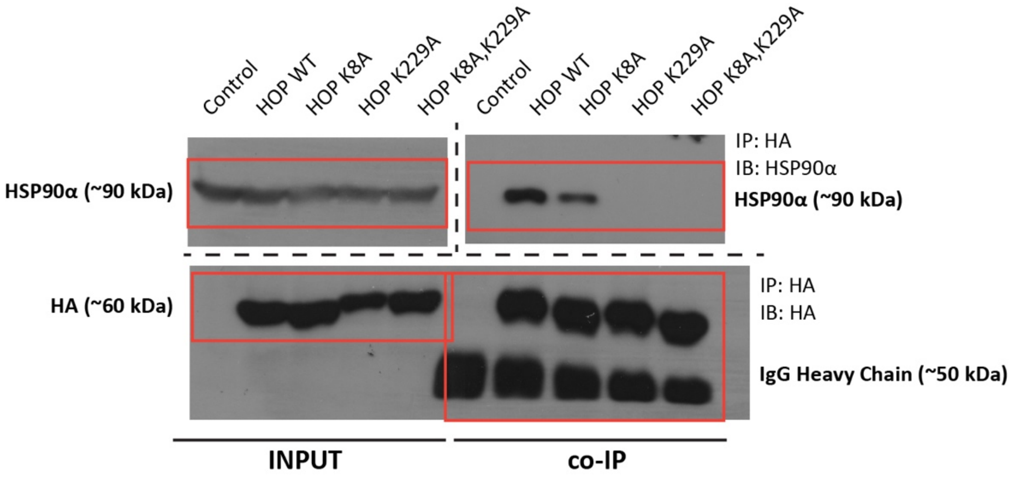

Figure S2. Uncropped original images of the immunoblots of Fig. 5. The nitrocellulose membranes were dissected as indicated by the dashed lines and incubated with different antibodies. Red boxes indicate image areas shown in the indicated panels.
